# High-resolution mapping of transcriptional dynamics across tissue development reveals a stable mRNA–tRNA interface

**DOI:** 10.1101/gr.176784.114

**Published:** 2014-11

**Authors:** Bianca M. Schmitt, Konrad L.M. Rudolph, Panagiota Karagianni, Nuno A. Fonseca, Robert J. White, Iannis Talianidis, Duncan T. Odom, John C. Marioni, Claudia Kutter

**Affiliations:** 1 University of Cambridge, Cancer Research UK Cambridge Institute, Cambridge, CB2 0RE, United Kingdom;; 2 European Molecular Biology Laboratory, European Bioinformatics Institute, Wellcome Trust Genome Campus, Hinxton, Cambridge, CB10 1SD, United Kingdom;; 3 B.S.R.C. Alexander Flemming, 16672, Vari, Athens, Greece;; 4 University of York, Department of Biology, Heslington, York, YO10 5DD, United Kingdom

## Abstract

The genetic code is an abstraction of how mRNA codons and tRNA anticodons molecularly interact during protein synthesis; the stability and regulation of this interaction remains largely unexplored. Here, we characterized the expression of mRNA and tRNA genes quantitatively at multiple time points in two developing mouse tissues. We discovered that mRNA codon pools are highly stable over development and simply reflect the genomic background; in contrast, precise regulation of tRNA gene families is required to create the corresponding tRNA transcriptomes. The dynamic regulation of tRNA genes during development is controlled in order to generate an anticodon pool that closely corresponds to messenger RNAs. Thus, across development, the pools of mRNA codons and tRNA anticodons are invariant and highly correlated, revealing a stable molecular interaction interlocking transcription and translation.

Transcription of the mammalian genome is divided among multiple RNA polymerases (Pol), each transcribing a nonoverlapping set of genes. Messenger RNAs (mRNAs) for protein-coding genes are synthesized by Pol II, while the genes encoding transfer RNAs (tRNAs) are transcribed by Pol III. The direct interaction of these transcripts produced by Pol II and Pol III is a vital step in the flow of genetic information, in which the triplet codons in mRNAs are selectively identified by their counterpart tRNA anticodons to direct protein synthesis. To explore the largely unknown regulatory mechanisms active at this mRNA–tRNA interface, we exploited the rapid and extensive changes in the transcriptome occurring among different developmental stages of mammalian organogenesis (Kyrmizi et al. 2006; Li et al. 2009; Kang et al. 2011; Lee et al. 2012; Liscovitch and Chechik 2013; Sunkin et al. 2013).

Conceptually, one possible mechanism to control protein abundance in developing tissues could be the deliberate mismatch of triplet codons in mRNAs and their corresponding tRNA anticodon isoacceptors (Brackley et al. 2011). In protozoa, this strategy is used to modulate the rate of translation of specific subsets of mRNAs containing a particular profile of codons (Horn 2008). Alternatively, if the large-scale changes in protein-coding transcriptomes result in a stable distribution of mRNA triplet codons, then deliberate changes in the population of tRNA anticodons could be used to fine-tune protein translation. Hypertranscription of tRNAs by Pol III has been observed in cancers (Winter et al. 2000; Pavon-Eternod et al. 2009, 2013), with recent work suggesting that differences in expression of specific tRNA genes may contribute to tumorigenesis by favoring translation of cancer-promoting mRNAs driving proliferation (Pavon-Eternod et al. 2009). It is unknown whether normal mammalian cells modulate tRNA gene expression to regulate information flow from mRNAs to protein synthesis.

We dissected the interdependencies of transcriptional and translational components in matched liver and whole brain tissue samples taken from C57BL/6J mice at eight developmental stages (from E9.5 to P29) on a genome-wide level by using RNA-sequencing (RNA-seq) and Pol III chromatin immunoprecipitation followed by sequencing (ChIP-seq) to quantify mRNA and tRNA gene expression levels, respectively (Fig. 1). Our quantitative analysis revealed that widely divergent protein-coding gene expression patterns contain a pool of codons that is stable and invariant. tRNA gene usage varies almost as extensively during development, yet also specifies a complementary pool of anticodons that is stable and invariant. We found a high correlation between these two pools, revealing that this key tRNA–mRNA interface is actively stabilized across mammalian development.

**Figure 1.**
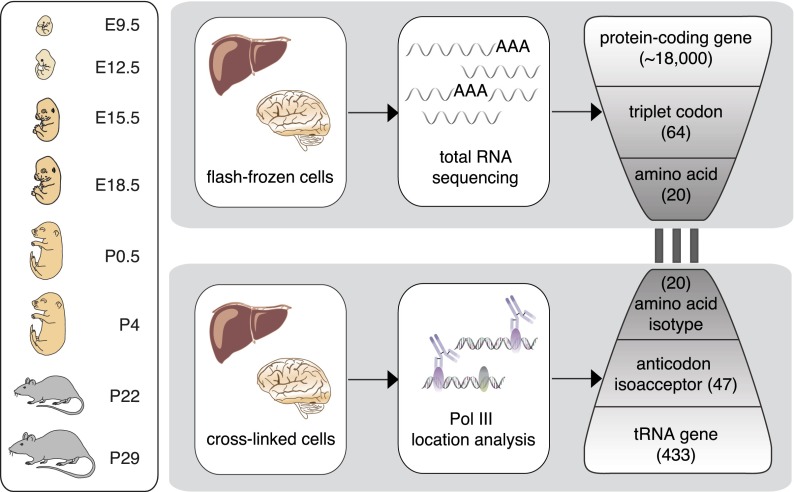
Transcriptome-wide analysis of protein-coding and tRNA genes during mouse organ development. Liver and brain tissues were isolated at eight mouse developmental stages. Tissue samples were flash-frozen for RNA-sequencing (RNA-seq) and cross-linked using formaldehyde to preserve protein–DNA interactions for ChIP-sequencing (ChIP-seq) of Pol III. Using the RNA-seq data, we calculated from all expressed protein-coding genes the frequencies of each triplet codon for all 64 possible codons and 20 amino acids. Similarly, Pol III binding to tRNA genes in the mouse genome was collapsed into 47 anticodon isoacceptor families and 20 amino acid isotypes (Methods). The bars linking RNA- and ChIP-seq data represent the three nucleotide interactions between codon and anticodon. Pol III occupancy was determined also in E9.5 (whole embryo) and E12.5 (head vs. remaining body).

## Results

### Mouse tissue development as a model system to study mRNA and tRNA gene regulation

Organogenesis during mouse development is a well-understood process (McLin and Zorn 2006; Bruneau 2008; Zorn and Wells 2009; Si-Tayeb et al. 2010; Kang et al. 2011). For example, the molecular landscape within the liver is known to undergo radical changes during development in response to shifts in the liver’s physiological functions during embryogenesis. During early development, the embryonic liver is a haematopoetic organ; at birth, the neonatal liver becomes the primary metabolic and detoxification organ (Si-Tayeb et al. 2010); at weaning, further metabolic pathways are up-regulated (Bohme et al. 1983; Girard et al. 1992). In the developing brain, coordinated gene expression changes in a heterogeneous collection of diverse cell types shape the functional specialization of specific regions in both embryonic and postnatal brains (Liscovitch and Chechik 2013; Sunkin et al. 2013).

To characterize changes in the mRNA and tRNA transcriptomes during development, we performed strand-specific, total RNA-seq, as well as ChIP-seq against Pol III in C57BL/6J mice in liver and brain at the following developmental stages: two embryonic (E15.5 and E18.5), two post-birth (P0.5 and P4), and immediately pre- and post-weaning (P22 and P29) stages (Fig. 1). For each experiment at each tissue and developmental stage, we performed two biological replicates that were highly correlated (Supplemental Figs. 1–3; Methods). This approach allowed us to quantify expression levels for protein-coding genes, as well as Pol III occupancy at every tRNA locus, which quantitatively captures the utilization of each tRNA gene (Barski et al. 2010; Moqtaderi et al. 2010; Oler et al. 2010; Kutter et al. 2011; Canella et al. 2012; Carriere et al. 2012; Renaud et al. 2014).

### Dynamic changes in protein-coding gene expression during mouse development

**Figure 2.**
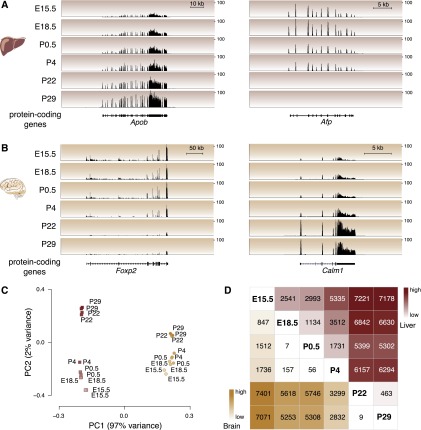
Protein-coding genes are differentially expressed in developing mouse liver and brain. Representative examples of protein-coding gene expression during development are (*A*) total RNA-seq reads mapping to *Apob* and *Afp* genes in liver and (*B*) *Foxp2* and *Calm1* genes in brain. The *y*-axis of each track specifies normalized read density. The scale bar shows length of genomic regions in kilobases (kb). (*C*) Factorial map of the principal component (PC) analysis of global protein-coding gene expression levels in liver (red) and brain (yellow) tissues. The proportion of variance explained by each principal component is indicated in parentheses. Color gradient indicates developmental stage (light: young; dark: old). (*D*) The intersection of the row/column for each developmental stage combination shows the number of differentially expressed protein-coding genes between the respective stages in liver (*top right* triangle) and brain (*bottom left* triangle) (0.1% FDR).

As expected, between stages we saw large-scale changes in the expression of protein-coding genes known to have different functions during liver and brain development (Li et al. 2009; Kang et al. 2011; Lee et al. 2012; Liscovitch and Chechik 2013). For instance, *Apob*, which is the primary apolipoprotein carrying low-density lipoproteins, is steadily up-regulated during development; in contrast, alpha fetoprotein (*Afp*), the fetal version of serum albumin, is down-regulated through development and replaced by its adult counterpart (Fig. 2A; Supplemental Table 1; Chen et al. 1997; Lee et al. 2012). By performing matched RNA-seq experiments during brain development, we observed similar dynamics of gene expression rewiring at the neural transcription factor *Foxp2*, where transcription decreases steadily after birth, and at the neurotransmitter calmodulin (*Calm1*), where transcription increases after birth (Fig. 2B; Supplemental Table 2; Huang et al. 2011; Tsui et al. 2013).

We then examined genome wide how protein-coding gene expression levels varied through development and between tissues. Using the set of protein-coding genes expressed in either liver or brain at any developmental stage, we calculated the pairwise correlation matrix and then performed principal components analysis (PCA) (Fig. 2C; Supplemental Fig. 4A). The vast majority (97%) of variation is explained by tissue identity (i.e., brain vs. liver), reflecting the dramatic differences in tissue-specific transcription that have been previously reported (Brawand et al. 2011). The large majority of the remaining variance (71%) orders the samples by developmental stage. Liver showed stronger differences during development than did brain, including between P4 and P22, consistent with the haematopoetic-to-metabolic changes occurring during liver development (Zorn and Wells 2009; Si-Tayeb et al. 2010). Comparison of all pairs of developmental stages revealed a steady increase in the number of differentially expressed protein-coding genes from early to later timepoints (Fig. 2D; Supplemental Tables 3, 4). The transcripts preferentially expressed during early development, either in brain or liver, revealed that these tissues were undergoing rapid cellular replication and expansion. In contrast, the transcripts up-regulated in adult reflected the mature biology of the tissues (Supplemental Tables 5, 6).

In summary, our quantitative and high-resolution gene expression profiles of liver and brain developmental stages revealed large-scale transcriptional rewiring in protein-coding genes, mirroring well-known aspects of developmental biology.

### Dynamic changes of tRNA gene expression during mouse development

We created a complementary data set by quantifying tRNA expression changes across development (Fig. 1; Supplemental Fig. 1B; Supplemental Table 7; Methods). tRNAs represent one of the largest gene families in mammalian genomes. Because several tRNA gene copies are identical in sequence, RNA-based methods (e.g., RNA-seq) alone are insufficient to determine the genomic location and rate of transcription of these genes. Pol III-binding to multicopy genes and importantly, to the unique sequence in the flanking regions has been established as a robust measure of tRNA gene usage and transcript abundance (Methods) (Barski et al. 2010; Moqtaderi et al. 2010; Oler et al. 2010; Kutter et al. 2011; Canella et al. 2012; Carriere et al. 2012; Renaud et al. 2014). From E15.5 to P29 in liver and brain, 311 of the 433 tRNA genes predicted by tRNAscan-SE (Schattner et al. 2005) were utilized (Supplemental Fig. 1B). Overall, the set of tRNA genes expressed during mouse development was similar to the tRNA genes identified as expressed in previous studies (Kutter et al. 2011; Canella et al. 2012; Carriere et al. 2012; Renaud et al. 2014). The overwhelming majority (93%, 290 of 311) of active tRNA genes resided in genomic clusters (Methods). The 311 tRNA genes identified in this study corresponded to 47 anticodon isoacceptor families, which represented all 20 amino acid isotope classes. Individual tRNA genes of a specific tRNA anticodon isoacceptor family tended to genomically cluster with another tRNA gene of the same family (Methods).

**Figure 3.**
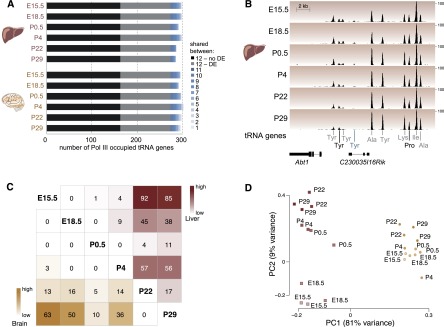
tRNA genes are differentially expressed during mouse development. (*A*) Stacked bar graph representing total number of expressed tRNA genes in developing mouse liver and brain tissue. Black (no differential expression) and gray (differential expression) represent number of tRNA genes expressed in all stages and tissues. In blue are tRNA genes that are shared between one and 11 stages. (*B*) Pol III binding to tRNA genes for the same genomic region during mouse liver development. Different colors of tRNA gene identifiers correspond to those used in *A*. The *y*-axis of each track specifies normalized read density. Scale bar shows length of genomic regions in kilobases (kb). (*C*) The intersection of the row/column for each developmental stage combination shows the number of differentially expressed tRNA genes in liver (*top right* triangle) and brain (*bottom left* triangle) (0.1% FDR). (*D*) Factorial map of the principal component (PC) analysis of tRNA gene expression levels in liver (red) and brain (yellow). The proportion of variance explained by each principal component is indicated in parentheses. Color gradient indicates developmental stage (light: young; dark: old).

A set of 272 tRNA genes was expressed in both tissues at all stages (Fig. 3A, Supplemental Fig. 5A, Supplemental Table 7). Within these 272 tRNA genes, we observed both a core set of tRNAs (*n* = 110, 40%) that showed no changes in Pol III occupancy at any stage of development, and a set of tRNA genes (*n* = 162, 60%) whose expression quantitatively changes during development. A smaller number of tRNA genes (*n* = 39) showed no Pol III occupancy in at least one stage of tissue development (Fig. 3A,B; Supplemental Fig. 5B).

We asked whether additional tRNA genes not identified post E15.5 may be expressed during very early developmental stages; we therefore profiled Pol III occupancy in E9.5 (whole embryo) and E12.5 (head vs. remaining body). In addition to the 311 tRNA genes, only 14 tRNA genes were newly identified as actively transcribed, all at low levels, at these earlier stages (Supplemental Fig. 6).

We identified the tRNA genes with altered expression levels during liver and brain development by using DESeq2 to compare pairs of timepoints (Fig. 3C; Methods). The largest number of changes was found between the embryonic and adult stages, with liver showing a higher number of differences than brain (up to 30% of liver and up to 20% of brain tRNA genes; Supplemental Tables 8, 9). PCA further revealed that tissue identity accounts for most (81%) of the total variance. The second component, explaining 46% of the remaining variance, clearly ordered samples by developmental stage (Fig. 3D). The liver samples showed a more pronounced variance between stages and stronger separation of pre- and post-birth samples than did brain. Thus, paralleling mRNA results, our data revealed that the changes in tRNA gene expression also reflect the pronounced functional shift in developing liver (Si-Tayeb et al. 2010).

In summary, we have discovered that developmental changes in tRNA transcription largely occur by altering the quantitative expression of a core set of just over 300 tRNA genes.

### Every mouse mRNA transcriptome encodes the same distribution of triplet codons and amino acids

**Figure 4.**
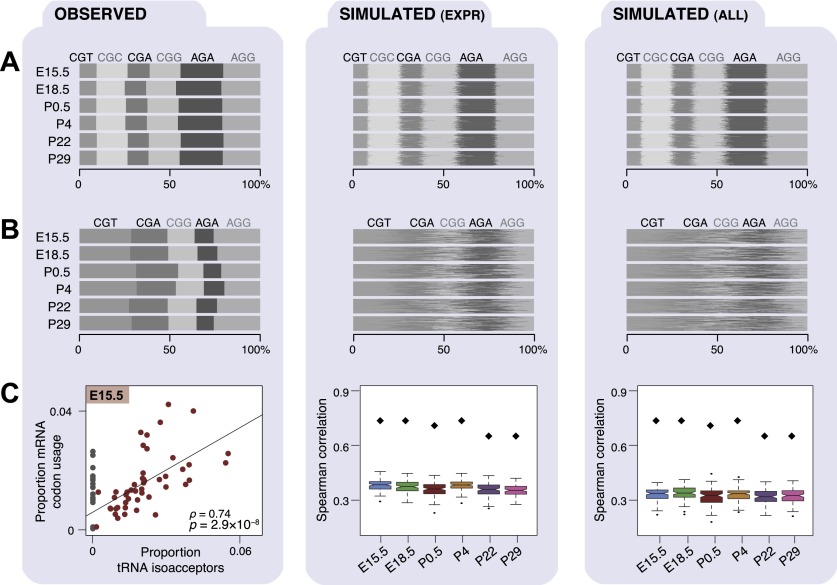
Codon and anticodon usage in transcriptomes across mouse development. Each panel (*A–C*) consists of three columns: experimentally observed data (*left*), simulated patterns of transcription randomized among either the expressed genes (*middle*), or all genomically encoded genes (*right*). Transcriptomes of each developmental stage were simulated 100 times (Methods). Proportional frequencies weighted by transcript expression are shown for arginine triplet codons as a bar plot (*A*), where gray shading is by triplet codon. Proportional frequencies weighted by Pol III binding are shown for arginine isoacceptors as a bar plot (*B*). (*C*) Plots show Spearman’s rank correlation coefficients (ρ) and *P*-values (*P*) of Pol III binding to tRNA isoacceptors (*x*-axis) and transcriptomic codon frequencies weighted by expression obtained from mRNA-seq data in E15.5 liver (experimentally observed data) and all six developmental stages (simulated data). Anticodon isoacceptors that are not encoded in the mouse genome (gray dots in *C*) were excluded from calculating the correlation coefficients. Observed correlations across all stages are indicated by black diamonds in plot *C*, *middle* and *left* panels.

Given the differences in mRNA expression levels observed during development, we investigated whether these changes create different distributions of triplet codons and amino acids. During translation, the coding sequence of each mRNA is read as a succession of 64 possible triplet codons, of which 61 correspond to 20 amino acids. We used our RNA-seq data to examine the abundance of each triplet codon and amino acid in the mRNA transcriptome, while accounting for transcript abundance in each developmental stage (Methods). Across the different developmental stages in both tissues, the frequencies of triplet codons (Fig. 4A, left; Supplemental Fig. 7) and encoded amino acids (Supplemental Fig. 8A, left) within mRNAs were highly stable (Spearman’s ρ ≥ 0.97 and Spearman’s ρ > 0.99, respectively) (Supplemental Figs. 4C,E, 9A,B). This pattern was also apparent when considering only genes that were highly or lowly expressed in individual stages (Supplemental Fig. 10A,B).

Overall amino acid usage is also stable among diverse mouse tissues, and even among homologous tissues from highly divergent mammals (Kutter et al. 2011). In mouse, adult liver, muscle, and testes have very different mRNA transcriptomes, which nevertheless contain a largely identical distribution of codon triplets and encoded amino acids, as do the different developmental stages of liver and brain that we reported above. We further found that the pattern of amino acid usage in all developmental stages is highly similar to the background distribution of triplet codons in the exonic sequences of about 21,000 protein-coding genes in the mouse genome (Supplemental Fig. 8A, left, gray contour; Methods).

We therefore asked whether the triplet codons present in any possible mouse transcriptome would follow the same triplet codon and amino acid distributions. We computationally created 100 artificial transcriptomes by shuffling the expression levels in each stage of development, first across all expressed genes (Fig. 4A, middle; Supplemental Fig. 8A, middle; Methods) and then over all annotated protein-coding genes (Fig. 4A, right; Supplemental Fig. 8A, right). The triplet codons and amino acids found in the simulated transcriptomes matched the background frequency.

Our data indicate that the stability of the triplet codon and amino acid distributions that we observed across development is, in fact, intrinsic to any possible transcriptome arising from the mouse genome.

### Stable isoacceptor anticodon abundance through development indicates tight regulation of tRNA gene expression

Since no differences in triplet codon and amino acid usage were observed in mRNA transcriptomes during development, we tested whether this was reflected in the availability of tRNA anticodon isoacceptors and amino acid isotypes. Relative utilization was calculated by summing the use of their component tRNA genes (Fig. 4B, left; Supplemental Figs. 9C,D, 11). The utilization of tRNA isoacceptors and isotypes was highly correlated between all developmental stages (Spearman’s ρ ≥ 0.96 and ρ ≥ 0.95 in liver and brain, respectively; Supplemental Fig. 4D,F).

We then asked whether the distribution of tRNA amino acid isotypes is similar to that found in the genomic background, as was seen for mRNAs above. In contrast to the tens of thousands of protein-coding genes, however, the total number of tRNA genes that can be used as a background in the mouse genome is 433 (Schattner et al. 2005). As for mRNAs, we created 100 artificial tRNA transcriptomes by shuffling the expression levels of each tRNA gene in each stage of development, first across the 311 expressed tRNA genes (Fig. 4B, middle) and then over all 433 annotated tRNA genes (Fig. 4B, right). We observed that the tRNA transcriptomes were substantially shifted from both sets of simulated tRNA transcriptomes, suggesting that they must be tightly regulated in order to create the stable pools of tRNAs observed across mouse development.

### mRNA triplet codon usage is highly correlated with tRNA anticodon isoacceptor abundance during development

tRNAs are the adapter molecules in the translational machinery that decode the triplet codons embedded in mRNA sequences. We explored this interface between transcription and translation by calculating the correlation between relative mRNA triplet codon usage and relative tRNA anticodon isoacceptor abundance. This analysis established whether the demand for triplet codons during mRNA translation is matched by the availability of corresponding tRNA anticodon isoacceptors. Omitting wobble pairings, we found a highly significant correlation between mRNA codon demand and corresponding tRNA anticodon availability in both tissues and at all developmental stages (Spearman-rank test [ρ] from 0.64 to 0.76, all *P* < 0.001), (Fig. 4C, left; Supplemental Fig. 12). We attempted to at least partially account for any effect that omitting the wobble position might have upon the measured proportions of tRNA isoacceptors (dos Reis et al. 2004) and obtained broadly comparable and highly statistically significant correlations (Spearman-rank test [ρ] from 0.49 to 0.64, all *P* < 0.001, Supplemental Fig. 13; Methods). The highly significant correlation persisted when considering mRNA amino acid usage and tRNA amino acid isotype abundance (all *P* < 0.001 [Spearman-rank test]; Supplemental Fig. 8, left; Supplemental Fig. 14).

Translational selection based upon differential usage of mRNA triplet codons is ubiquitous in prokaryotes, particularly in highly expressed and translated genes (Supek and Smuc 2010). To investigate whether a similar translational selection mechanism exists during mouse development, we correlated mRNA triplet codon usage of highly and lowly expressed genes to tRNA anticodon isoacceptor abundance for each stage. The distributions of correlation values over all developmental stages are similar between (1) highly and lowly expressed gene sets and (2) in comparing these with all expressed genes (Supplemental Fig. 10C–E).

In the simulated transcriptomes, the correlation of mRNA triplet codon usage and tRNA anticodon isoacceptor abundance was appreciably lower than in the empirical data (all Spearman’s ρ < 0.45) (Fig. 4C, middle and right) providing further evidence that regulation is required to create the appropriate tRNA transcriptome. At the amino acid level, correlations in the mRNA and tRNA transcriptomes remained lower than the empirically determined correlations when considering all genes; no difference was observed for simulations using only expressed genes (Spearman’s ρ < 0.90; Supplemental Fig. 8C, middle and right).

Thus, the supply of tRNA gene transcripts is controlled, creating pools of anticodon isoacceptors that track the demand of the triplet codons found in mRNA transcriptomes.

### tRNA anticodon isoacceptor families are transcriptionally compensated across development

The expression levels of individual tRNA genes can vary substantially during development and this variation can distinguish specific developmental stages. We considered the possibility that the expression differences of individual tRNA genes are driven by local features, such as the upstream sequence composition or the expression of nearby mRNA genes.

First, we chose to analyze the *cis* regulatory sequences within 500 bases of the transcription start site of tRNA genes. We did this since prior studies have shown that sequence variation in internal regulatory sequences of tRNA genes have no clear relationship with their expression levels (Oler et al. 2010; Canella et al. 2012). We searched for regulatory sequence elements acting in *cis* that could direct the Pol III recruitment to tRNA genes as described previously (Giuliodori et al. 2003; Bloom-Ackermann et al. 2014). To investigate the differences in Pol III binding for every pair of timepoints, we used MEME (Bailey and Elkan 1994) and TOMTOM (Gupta et al. 2007) to search for enrichment of specific motifs in the 500-bp upstream regions of differentially expressed tRNAs, using stably expressed tRNA genes as the background (Supplemental Table 10; Methods). No strong enrichment of specific motifs was observed in the upstream regions of differentially expressed tRNA genes, suggesting that regulatory sequences in the flanking regions do not explain differences in tRNA gene expression.

Second, we considered whether transcription of proximal protein-coding genes could influence nearby tRNA transcription (White 2011). We asked whether significant changes of individual tRNA genes’ utilization between stages were accompanied by changes in expression of neighboring protein-coding genes. No consistent colocalization effects were observed (Supplemental Fig. 15; Methods).

Third, we investigated whether the activity of tRNA transcription is mediated via chromatin or other *cis*-regulatory sequences. Previous studies reported that active chromatin coincides with active transcription of Pol III genes (Barski et al. 2010; Oler et al. 2010). We therefore used previously published data (Shen et al. 2012) to compare the occurrence of three histone marks associated with transcriptionally active chromatin modifications, histone H3 lysine 27 acetylation (H3K27ac), H3 lysine 4 tri- (H3K4me3) and monomethylation (H3K4me1), Pol II, and the insulator-binding protein CCCTC-binding factor (CTCF) at genomic localizations 0.1, 0.5, and 1.0 kb near differentially expressed tRNA genes (Shen et al. 2012; Methods). In mouse liver, we found a significant association between H3K27ac levels 0.5 and 1.0 kb around tRNA genes that are differentially expressed between E15.5 and P29 (Fisher’s exact test, *P*-value < 10^−4^) (Supplemental Table 11). H3K4me3 and Pol II showed a less significant association, while H3K4me1 and CTCF were not associated with differentially expressed tRNA genes between these two developmental stages (Supplemental Table 11). These results suggest that differential expression of tRNA genes during mouse development can be facilitated in part by accessibility to active chromatin.

Although there are multiple tRNA genes with varying levels of expression for most isoacceptors, the collective relative expression of the tRNA genes within each family is stable throughout development (Fig. 4B, left). This raised the possibility that the expression of individual tRNA genes within each isoacceptor family might vary randomly during development. If this were the case, no systematic correlation in expression levels between genes within the same tRNA anticodon isoacceptor family during development would be expected.

**Figure 5.**
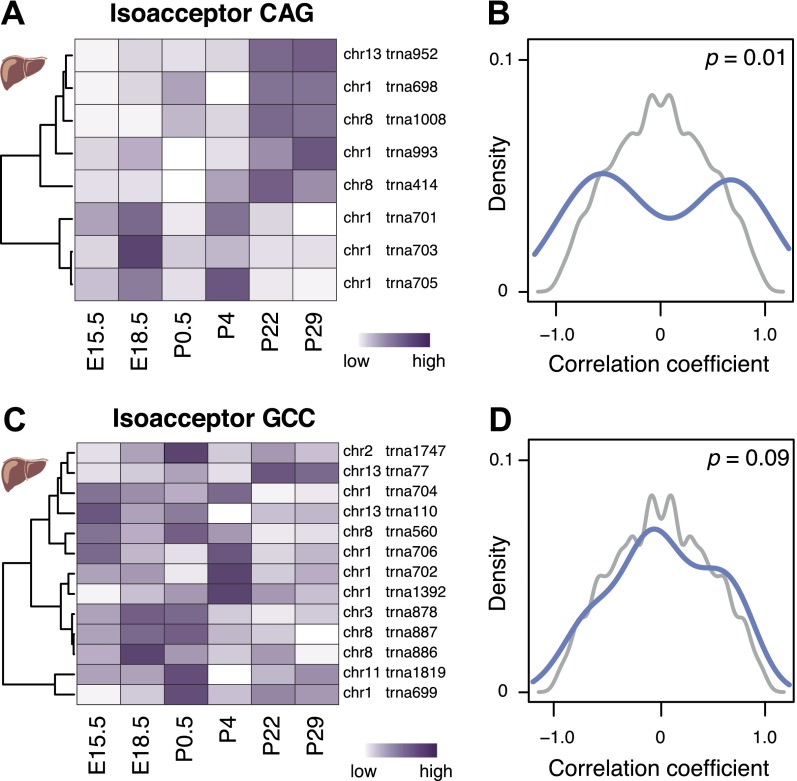
tRNA gene expression is compensated on the isoacceptor level during mouse development. Anticodon isoacceptor Leu(CAG) (*A*) and Gly(GCC) (*C*) illustrate a strong and weak correlation of tRNA gene expression level, respectively. Each row of the heatmap represents relative expression levels of tRNA genes across different developing mouse liver stages (white, low = 0; purple, high = 1). Density plots (*B*,*D*) represent the distribution of pairwise correlation coefficients between each tRNA gene’s expression levels during mouse liver development for anticodon isoacceptor (*B*) Leu(CAG) and (*D*) Gly(GCC) (blue). Background distributions (gray) are derived by permuting the order of stages when computing the pairwise correlation between tRNA genes. *P*-values (*P*, χ^2^-test) are reported in *top right* of each panel.

We therefore searched for evidence of gene–gene correlations within each family containing more than five tRNA genes (Methods) that deviated systematically from a background distribution, generated by permuting the order of the stages for each gene. This background yielded correlation coefficients that followed a unimodal distribution centered around zero. Of the 27 isoacceptor families containing six or more tRNA genes, 16 showed a bimodal distribution of correlation coefficients (59%; *P* < 0.0199, FDR corrected) (Fig. 5; Supplemental Table 12; Methods), pointing to the existence of two distinct expression clusters of genes with high correlation within each expression cluster and negative correlation between expression clusters (Fig. 5A,B). There is no significant difference between these two groups of isoacceptor families with regard to the degree of genomic clustering of their respective tRNA gene members. Thus, the expression levels of tRNA genes for the majority of isoacceptor families are coupled, such that a decrease in the expression of one gene can be compensated for by an increase in the expression of another.

Our data suggest that the local genomic environment cannot explain the specific regulatory mechanism underlying changes in tRNA gene utilization during development. Nevertheless, within many isoacceptor families, we observe striking compensation in expression levels between tRNA genes. Our current understanding of mammalian tRNA gene regulation has no mechanism that could account for such coordinated and selective crosstalk (White 2011).

## Discussion

The process of organ development requires precise stage-specific coordination of gene expression, leading to highly variable pools of mRNAs. The widespread changes in protein-coding gene expression occurring during mammalian development have been previously explored using gene-specific (Wutz et al. 1997; Kyrmizi et al. 2006; Mallo and Alonso 2013) and transcriptome-wide approaches (Bruneau 2008; Kang et al. 2011; Nord et al. 2013). Here, we confirmed that liver and brain development are accompanied by thousands of protein-coding transcript changes, which accurately reflect the tissue and stage identity of all samples. Our gene expression data sets complement recent studies mapping enhancer deployment during mouse development (Cotney et al. 2013; Nord et al. 2013). The observed number of differentially expressed genes with respect to E15.5 gradually increased over developmental time, and corresponded to tissue and developmental stage-specific functions. Just over a third of genes in both tissues are differentially expressed between any two stages, consistent with previous results from microarray-based analyses (Li et al. 2009).

In prokaryotes, large-scale gene expression changes can cause shifts of mRNA triplet codon usages that impact translation rates (Tuller et al. 2010); such a mechanism has been suggested for highly expressed mouse and human transcripts (Plotkin et al. 2004; Lavner and Kotlar 2005; Dittmar et al. 2006). In our transcriptome-wide approach, we did not find differential codon usage between highly and lowly expressed protein-coding genes in each developmental stage; however, we cannot fully exclude subtle fine-tuning of triplet codon usage of selected protein-coding genes that are tissue- and developmental stage specifically expressed (Plotkin et al. 2004). Our results indicated that the triplet codons within mRNA transcripts and thus the collective amino acid demand placed on translational machineries remain largely invariant across mouse development. Remarkably, this stable distribution of triplet codon usage is found in every developmental stage and tissue we examined, as well as randomly generated transcriptomes created from the mouse genome. The stability of the triplet codon usage is also apparent among analogous adult tissues in divergent species across 180 MY of evolution (Kutter et al. 2011), suggesting a conserved transcriptomic feature in mammals. In sum, the same distribution of triplet codons that must be translated by tRNA anticodons is consistently and robustly found within transcriptomes throughout development and across evolution.

These triplet codons in mRNA transcripts form transient, noncovalent hydrogen bonds with decoding tRNAs, creating the molecular interface that connects transcription and translation (Fig. 1). In principle, protein synthesis could be influenced by tRNA abundance if anticodon isoacceptors and amino acid isotypes deviate from the levels of their mRNA counterparts (Horn 2008; Brackley et al. 2011). However, mammalian development does not appear to exploit this regulatory strategy as we have discovered that across mouse organogenesis, mRNA codons and tRNA anticodons are highly correlated at each developmental stage. Instead, there is a molecular equilibrium between the populations of codons found in expressed mRNAs and anticodons found in expressed tRNAs. In fact, this correlation may be higher if considering post-transcriptional modifications such as tRNA-dependent adenosine deaminases (Novoa et al. 2012). This molecular equilibrium can prevent a potential bottleneck when tRNAs facilitate translation of mRNAs into proteins and hence ensures optimal translational efficiencies.

Such stability in anticodons in the pool of expressed tRNAs might suggest a model where the tRNA genes transcribed by Pol III do not vary across mouse development. This model would predict stable Pol III occupancy, which would allow the cell to maintain high efficiency in translation without having to adjust components of the translational machinery. Surprisingly, we found that, similar to the changes in the protein-coding transcriptome, up to 30% of tRNA genes in liver and up to 20% in brain are differentially expressed between developmental stages. However, the vast majority of differences in tRNA expression are adjustments to the level of expression of individual tRNA genes, and are not de novo activation or inactivation events.

Molecular characteristics that appear to have little or no influence in distinguishing the differentially utilized tRNA genes include (1) spatial clustering in the genome, (2) tRNA gene copy number, (3) isoacceptor and isotype frequency, (4) *cis*-regulatory elements proximal to tRNA genes, and (5) the expression of nearby protein-coding genes. In contrast, we discovered that tRNA expression may be facilitated in part by specific chromatin states associated with active transcription, including the active enhancer mark of H3K27ac and, to a lesser extent, H3K4me3. Further, the expression of the anticodon isoacceptor families, relative to each other, was stable across mammalian development. In yeast, tRNA genes can compensate for changing levels of expression of another member of their isoacceptor family, an effect demonstrated by systematic deletion of tRNA genes (Yona et al. 2013; Bloom-Ackermann et al. 2014). Our data are a first direct indication that a compensatory mechanism must also operate dynamically in mammals to stabilize the collective expression of a given isoacceptor family. Because tRNA gene usage changes throughout development, but nevertheless produces stable isoacceptor anticodon expression, feedback mechanisms must exist to maintain the isoacceptor anticodon’s steady state. Supporting this model, a substantial fraction of anticodon isoacceptor families demonstrate a dramatic and coordinated regulation among family members (Fig. 5A).

Across mammalian development, protein-coding and tRNA gene expression vary widely, require different transcriptional machineries, and are controlled by distinct mechanisms. Our data indicate that new models of tRNA gene regulation are required to explain how both converge to generate a highly stable molecular interface, functionally interlocking transcription and translation.

## Methods

### Tissue preparation

C57BL/6J mice were bred and housed in the Biological Resources Unit under UK Home Office licensing. Liver and whole brain tissues were dissected from mice at different developmental stages. Whole embryos were collected at E9.5 and tissues from heads versus remaining body at E12.5. Tissues from embryonic litters were pooled. At least two independent biological samples were obtained for each tissue and stage. Tissues were either post-mortem cross-linked or fresh-frozen in liquid nitrogen.

### Chromatin immunoprecipitation followed by high-throughput sequencing (ChIP-seq) library preparation

ChIP-seq assays were performed as previously described (Kutter et al. 2011). Protein-bound DNA was immunoprecipitated with an antibody against Pol III subunit RPC1/155, a component that is involved in active tRNA gene transcription. Immunoprecipitated DNA was end-repaired, A-tailed, and single-end Illumina sequencing adapters ligated before 18 cycles of PCR amplification. A total of 200- to 300-bp DNA fragments were selected and 36-bp single-end reads sequenced on an Illumina Genome Analyzer IIx or HiSeq 2000 according to the manufacturer’s instructions.

### Total RNA-sequencing (RNA-seq) library preparation

Total RNA was extracted from livers and brains of all stages and prepared for sequencing (in biological duplicates). RNA samples were ribosomal RNA depleted (RiboZero, Epicenter). Strand-specific libraries were prepared using dUTPs (Kutter et al. 2012) and multiplexed (Illumina TruSeq kit), prior to 75-bp paired-end sequencing on an Illumina HiSeq 2000, according to the manufacturer’s instructions.

### ChIP-seq analysis

Pol III ChIP-seq libraries were mapped to the mouse reference genome (NCBIM37) using BWA version 0.5.9-r16 (Li and Durbin 2009) with default parameters. The genomic locations of tRNA genes were identified by tRNAscan-SE version 1.21 (Lowe and Eddy 1997). Mitochondrial tRNA was excluded from the analysis. Because tRNA genes are frequently duplicated in the genome (Lowe and Eddy 1997), reads mapping equally well to multiple genomic locations were reallocated probabilistically to a single location, as described previously (Kutter et al. 2011). Reads with more than 20 matches were discarded. Expression of tRNA genes was determined by counting reads at each tRNA gene locus and ±100-bp flanking region. A tRNA gene was defined as expressed if at least 10 reads were mapped to it in both biological replicates for at least one tissue–timepoint combination. All subsequent analysis was performed using only this set of genes, excluding selenocysteine, except where noted otherwise.

### RNA-seq analysis

RNA-seq libraries were analyzed with iRAP (Fonseca et al. 2014), using TopHat2 (Kim et al. 2013) to map reads to the reference genome (NCBIM37) and HTSeq-count (Anders et al. 2014) to assign reads to the Ensembl release 67 gene annotation (Flicek et al. 2014) using default parameters, and by excluding mitochondrial and sex chromsome encoded genes.

### Differential expression (DE)

For both the mRNA and tRNA data sets, we used DESeq2 (Love et al. 2014) to identify genes differentially expressed between pairs of developmental stages with a Benjamini-Hochberg corrected *P*-value < 0.001.

### Principal components analysis (PCA)

For both the mRNA and tRNA data sets, PCA was applied to the matrix of pairwise Spearman rank correlations.

### Codon usage

First, for every gene, the number of occurrences of each codon in the longest annotated transcript was determined and this value was multiplied by the gene’s expression (normalized for transcript length). Next, the overall usage of each codon for each library was obtained by summing these values across all genes. A similar analysis was performed for amino acids. Subsequently, relative codon and amino acid usage (excluding selenocysteine) were calculated as
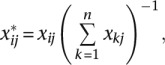
where 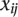 is the triplet codon (or amino acid) usage for triplet codon (or amino acid) 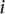 in stage 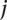, and 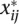 is the relative usage. Spearman’s rank correlation was used to establish the correlation between relative (1) codon and (2) amino acid usage on the protein-coding gene level with the abundance of tRNAs coding for matching (1) anticodon isoacceptors and (2) amino acid isotypes.

### Codon and anticodon background sampling

We used our library-size normalized RNA-seq and Pol III ChIP-seq data to simulate background distributions in liver and brain for each specific developmental stage. We randomly rearranged the expression values across genes for (1) the expressed and (2) all genomically annotated protein-coding and tRNA genes. For each developmental stage, we created 100 such random background distributions. We then calculated triplet codon and amino acid usage, as well as isoacceptor and isotype abundance, for the rearranged protein-coding RNA and tRNA expression distributions. We determined the mean for each of the 100 shuffled triplet codon and amino acid distributions and calculated their Spearman correlation coefficient with each of the 100 shuffled isoacceptor and isotype distributions.

### Wobble interaction correction

To estimate the influence of wobble base pairing on the codon–anticodon correlations, we first identified the 15 “orphan” mRNA triplet codons without a matching tRNA anticodon. These triplet codons are usually decoded by a closely related tRNA isoacceptor of the same isotype class (dos Reis et al. 2004). We matched these closely related tRNA isoacceptors to the orphan mRNA triplet codon. This resulted in some anticodon isoacceptors that can recognize more than one codon. In such cases we weighted the tRNA abundance by using the complementary mRNA-seq data: We estimated what fraction of the wobbling tRNA anticodon is most likely redirected to decode each matching mRNA triplet codon and accordingly divided its cumulative anticodon isoacceptor abundance between its matched mRNA triplet codons. We then calculated Spearman’s rank correlation between the weighted tRNA anticodon isoacceptor abundances and mRNA triplet codon usage.

### Motif analysis

The sequences of the 500-bp upstream regions of differentially expressed tRNA genes between all pairwise stages in each tissue from the forward and reverse strand were cleaned of low-complexity regions using the “dust” application. A first-order Markov model built from the upstream regions of all nondifferentially expressed tRNAs in the appropriate stage–stage contrast was used as background. Motif enrichment analysis in the sequences was conducted with MEME (Bailey and Elkan 1994), configured to search for zero or one occurrences of a motif per sequence, up to a maximum of three distinct motifs, with a minimum motif size of 6 bp. Subsequently, TOMTOM (Gupta et al. 2007) was used to interrogate the MEME output using “JASPAR_CORE_2009_vertebrates” and “uniprobe_mouse” as input databases. A minimum overlap of 5 bp with an *E*-value threshold of 10 was required.

### Colocalization

A test for colocalization of the largest set of differentially expressed tRNA genes and differentially expressed mRNA genes was performed between developmental stages (E15.5/P22 in liver and P4/P29 in brain). For each up-regulated tRNA gene 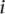, we counted the number of up-regulated protein-coding genes, 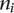, and total number of protein-coding genes, 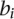, in the same genomic region of varying window sizes (10 kb, 50 kb, and 100 kb), which allowed us to compute the ratio 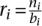. We repeated this analysis for nondifferentially expressed tRNA genes. A Kolmogorov-Smirnov test was performed to assess whether the distribution of ratios of up-regulated protein-coding genes was significantly different in the vicinity of up-regulated tRNA genes from that in the vicinity of nondifferentially expressed tRNA genes with varying significance thresholds (0.1, 0.05, and 0.01).

### Chromatin association

Publicly available ChIP-seq data of histone marks (Gene Expression Omnibus [GEO] accession GSE29184) associated with genomic regions at promoters and enhancers (H3K4me3, H3K4me1, H3K27ac), Pol II, and an insulator (CTCF) (Shen et al. 2012) were used to assess whether any of these marks were associated (Fisher’s exact test) with (1) active versus inactive tRNA genes in embryonic (E15.5) and adult (P29); and (2) differentially expressed tRNA genes between E15.5 and P29 in mouse liver and brain tissues. Occurrence of these chromatin marks was measured 0.1, 0.5, and 1 kb upstream of and downstream from tRNA genes. Our embryonic (E15.5) and adult (P29) Pol III data were complemented with embryonic (E14.5) and adult (P56) ChIP-seq data as different developmental time points were selected in our and in the Shen et al. (2012) study. Likewise, our brain P29 data were compared with P56 data by merging “cortex” and “cerebellum” ChIP-seq data from Shen et al. (2012).

### Compensation

For each isoacceptor that is encoded by more than two tRNA genes, we calculated Spearman’s rank correlation (across developmental stages) between the expression values of each pair of its corresponding tRNA genes. For the same set of genes, we calculated a null set of correlations as follows: all possible pairwise Spearman correlations obtainable by permuting the order of the developmental stages for each pair of genes. Next, we used the χ^2^-test to investigate whether there was a significant difference between the background and the observed correlation distributions. We reported the Bonferroni-corrected *P*-value for the 27 isoacceptor families with six or more genes, since isoacceptor families with less than six genes did not have enough points for meaningful interpretation.

### Genomic clusters

We defined 69 clusters of all genomically annotated tRNA genes that lie within 7.5 kb of each other. We counted how many active tRNA genes of an isoacceptor family colocalized in a genomic cluster with tRNA genes of the same isoacceptor family. We calculated the fraction of tRNA genes for each isoacceptor family belonging to a genomic cluster. In order to test whether genes in isoacceptor families tend to genomically colocalize more than expected by chance, we randomly assigned tRNA genes to isoacceptor families (preserving the actual isoacceptor family gene numbers) 1000 times. We then tested whether the mean percentage of clustering tRNA genes per isoacceptor family differed from the mean percentage expected by random by using a binomial test. Finally, we tested whether there was a difference in these percentages between isoacceptor families that show evidence for compensation, and isoacceptor families that show no such evidence by applying a χ^2^-test.

### Code availability

The code for the analysis is available in the Supplemental Material under SourceCode.zip and SourceCode_dataprocessing.zip and online at http://github.com/klmr/trna and http://github.com/klmr/trna-chip-pipeline, with the exception of the ChIP-seq read reallocation tool, which was kindly provided by Gordon Brown.

## Data access

Pol III ChIP-seq and RNA-seq data from this study have been submitted to the ArrayExpress database (http://www.ebi.ac.uk/arrayexpress/) under accession numbers E-MTAB-2326 and E-MTAB-2328, respectively. Additional data are available in the Supplemental Material under SupplementalData.zip and at http://dx.doi.org/10.6084/m9.figshare.942513.
